# Elevated cytokine levels in vitreous as biomarkers of disease severity in infectious endophthalmitis

**DOI:** 10.1371/journal.pone.0205292

**Published:** 2018-10-08

**Authors:** Dhanshree Deshmukh, Moumita Chakrabarti, Rajagopalaboopathi Jayasudha, Mohammed Hasnat Ali, Mudit Tyagi, Savitri Sharma, Joveeta Joseph

**Affiliations:** 1 Jhaveri Microbiology Centre, Brien Holden Eye Research Centre, L. V. Prasad Eye Institute, Hyderabad, Telangana, India; 2 Centre for Clinical Epidemiology and Biostatistics, L. V. Prasad Eye Institute, Hyderabad, India; 3 Smt. Kannuri Santhamma Centre for Vitreoretinal Diseases, L V Prasad Eye Institute, Kallam Anji Reddy Campus, Hyderabad, Telangana, India; University of Bern, SWITZERLAND

## Abstract

**Purpose:**

To investigate the immunopathogenesis of endophthalmitis, and determine if cytokine profiles could serve as biomarkers of disease severity in infectious endophthalmitis.

**Materials and methods:**

Vitreous samples of 46 patients clinically diagnosed as endophthalmitis (of which 25 were culture positive) and 20 non-infectious controls from patients with Retinal Detachment (RD) or diabetic retinopathy were included in the study. The cytokine and chemokine expression patterns of 40 immune mediators including 6 antiinflammatory cytokines, 15 proinflammatory cytokines, 9 Growth factors and 10 proinflammatory chemokines in the vitreous were were analyzed by multiplex cytokine immunoassay. In addition, significant immune mediators were correlated with initial and final visual acuity (VA).

**Results:**

Our results demonstrated elevated expression of 16 mediators such as GCSF, GRO, IFN-γ, IL-1α, IL-1β, IL-1 RA, IL-6, IL-8, IP-10, MCP-1, MCP-3, MIP-1α, IL-1β, TGF-α, TNF-α in patients with culture positive endophthalmitis. Cytokine profile expression significantly differed between patients with proven endophthalmitis and the non-infectious controls in heat map analysis. PCoA plot indicated five mediators (IL-1RA, IL-6, IL-8, GRO, G-CSF) as biomarkers that could be Independent Predictors of Disease especially in culture negative cases. Correlation of cytokines with VA revealed strong association between the initial VA and intraocular levels of TGF-α, IL-1β and IL-8 but there was no correlation with the severity or visual outcome of infection.

**Conclusion:**

In comparison to non-infectious ocular conditions, the pathogenesis of infectious endophthalmitis correlates with increased expression levels of IL-1RA, IL-6, IL-8, GRO, G-CSF. Understanding cytokine profiles in culture negative endophthalmitis patients could aid in therapy in non-responders to empirical antibiotic therapy.

## Introduction

Endophthalmitis continues to be a rare, but potentially sight threatening complication following ocular surgery, penetrating ocular trauma or systemic infection [1,2]. Risk factors include the age and immune status of the patient, condition of the eye upon presentation, the infecting organism’s virulence, antibiotic susceptibility profile, and the time between injury/surgery and therapy. While the role of intravitreal injection of antibiotics has been proven in the treatment of infectious endophthalmitis, the beneficial role of steroid administration is controversial and it remains so even today. In addition, damage can occur not only from toxins produced by the invading organisms but also by the inflammatory mediators of the immune response [3]. Overall, regardless of the source of infection, clinicians must take into account a myriad of unique challenges posed by the blood- ocular barrier, in order to protect the vision of patients with infectious endophthalmitis. There have some recent studies on the levels of various inflammatory mediators as markers for the activity and severity of ocular inflammation in aqueous and vitreous fluids [3–8]. While the role of certain immune mediators like tumor necrosis factor alpha (TNF-α) and interferon gamma (IFN-γ), have been studied in the development of endophthalmitis in animal models, very few expression studies have been carried out in humans suffering from infectious endophthalmitis [5,8]. Understanding the role of cytokines in infectious endophthalmitis may lead to advances in diagnosis and treatment.

Presumed infectious but culture negative endophthalmitis however is a challenging clinical entity, both diagnostically and therapeutically and clinicians often must treat the eye empirically. We hypothesized that comprehensive profiling of levels of multiple cytokines would provide greater insight into their utility for staging patients with endophthalmitis, and these cytokine profiles could be used as a biomarker and help modulate the treatment of endophthalmitis, especially when the etiologic basis of culture-negative endophthalmitis remains unclear. In this study, we compared the inflammatory mediators in vitreous samples of infectious endophthalmitis (culture positive and culture negative) patients and non-infectious controls to identify the molecules involved in the immunopathogenesis of endophthalmits, and determined whether specific cytokine profiles could be diagnostic in culture negative endophthalmitis cases and correlated them with the final visual acuity to check for predictors of outcome.

## Methods

### Subjects

This study was a prospective study. Patients were selected with a clinical diagnosis consistent with infectious endophthalmitis seen at the Retina clinic of the L V Prasad Eye Institute between June 2017 and December 2017. A written informed consent was obtained from each participant prior to their enrolment in the study and in case of minors, the written informed consent was obtained from their parents or legal guardians. Patients were excluded if vitreous gel (undiluted) sample was inadequate after routine microbiology workup. The study population included 46 patients diagnosed with infectious endophthalmitis of which 25 were culture positive and 21 were culture negative, in addition to 20 non-infectious controls. For the control group, vitreous gel (undiluted vitreous) were collected from a group of heterogenous patients undergoing posterior vitrectomy for non-infectious diseases (Retinal Detachment (RD), Diabetic Retinopathy (DR)) during the study period. In the test group, we had excluded cases which had a suspicious diagnosis or where the clinical characteristics were ambiguous. All patients diagnosed underwent complete ophthalmological examinations, including slit-lamp biomicroscopy, B-scan, visual acuity recordings and later underwent pars plana vitrectomy within 1 to 5 days after presentation. All cases (culture positive and culture negative) had clinical features suggestive of endophthalmitis like intense AC inflammation, extensive vitreous exudates, corneal infiltrates or a lens abscess. They were all clinically diagnosed and treated on lines of endophthalmitis. The vitreous gel from both patients and controls obtained during vitrectomy were collected aseptically in and sent to the laboratory for microbiological work up, of which 50 microliters was transferred into a presterilized microcentrifuge tube and stored at -80°C for further analysis.

### Microbiology

Vitreous samples from patients were investigated using institutional protocol as described earlier [9]. Briefly, the sample was inoculated directly onto blood agar, chocolate agar, Sabouraud dextrose agar, potato dextrose agar, thioglycollate broth, and brain-heart infusion broth. All media were incubated at 37°C except Sabouraud dextrose agar and potato dextrose agar, which were incubated at 25–27°C for a period of 7 days. One blood agar was incubated for anaerobic culture while all other media were incubated aerobically. A culture was considered positive when there was growth of the same organism on two or more media, and/or confluent growth at the site of inoculation on one solid medium, and/or growth in one medium with consistent microscopy findings. In case of positive bacterial cultures, the bacteria were further subjected to identification and antibiotic susceptibility testing. The bacterial isolates were identified by Vitek 2 (bioMérieux, France) after confirming by biochemical tests. Fungal species were however identified based on their sporulation and growth characteristics.

### Multiplex immunoassay

Fifty microliters of diluted (1:3) vitreous samples from both infectious and control subjects were analyzed for 40 human vitreous humor mediators (Table 1) by multiplex immunoassay using the Milliplex MAP kit from Merck Life Sciences and tested on the Luminex 200 System with xPONENT 3.1, Luminex Corporation, Austin Texas. The kit was a commercially available human cytokine/chemokine magnetic bead panel kit (Milliplex MAP, Millipore, Billerica, MA, USA) of 40 immune mediators as mentioned in Table 1. The analysis procedure was conducted according to the manufacturer’s instructions. Briefly, 50μl of 1:3 dilution of vitreous sample was centrifuged at 15000 rpm for 10min at 4°C and 25 μL of each sample and different concentrations of each cytokine standard were added to 50 μL antibody-conjugated beads in a 96-well filter plate. Following incubation with primary and secondary antibody, the plate was washed and analyzed using a BD Bead Array Reader. The concentrations of the cytokines were then calculated from a standard curve for each cytokine.

**Table 1:** Immune mediators analyzed in the study.

S. No	Immune mediator	
**Pro-inflammatory cytokines**		
1	IFNα2	Interferon alpha-2
2	IFN-γ	Interferon gamma
3	IL-12p40	Interleukin-12 subunit p40
4	IL-12p70	Interleukin-12 subunit p70
5	IL-15	Interleukin-15
6	sCD40L	Soluble CD40-ligand
7	IL-17A	Interleukin-17A
8	IL-1α	Interleukin 1 alpha
9	IL-1β	Interleukin 1 beta
10	IL-2	Interleukin-2
11	IL-3	Interleukin-3
12	IL-6	Interleukin-6
13	IL-7	Interleukin-7
14	TNFα	Tumor necrosis factor alpha
15	TNF-β	Tumor necrosis factor beta
**Pro-inflammatory chemokines**		
16	Eotaxin	Eotaxin
17	GRO	Growth-regulated Oncogene
18	MCP-1	Monocyte chemoattractant protein-1
19	MCP3	Monocyte chemotactic protein-3
20	RANTES	Regulated on Activation Normal T Cell Expressed and Secreted
21	IL-8	Interleukin-8
22	MDC	Macrophage-derived chemokine
23	IP-10	Interferon gamma-induced protein 10
24	MIP-1β	Macrophage Inflammatory Proteins 1 beta
25	MIP-1α	Macrophage Inflammatory Proteins 1 alpha
**Anti-inflammatory mediators**		
26	IL-10	Interleukin-10
27	IL-13	Interleukin-13
28	IL-1RA	Interleukin-1 receptor antagonist
29	IL-9	Interleukin-9
30	IL-4	Interleukin-4
31	IL-5	Interleukin-5
**Growth factors**		
32	EGF	Epidermal growth factor
33	FGF2	Fibroblast Growth Factor 2
34	TGF-α	Transforming growth factor alpha
35	G-CSF	Granulocyte colony stimulating factor
36	FLT3L	FMS-like tyrosine kinase 3 ligand
37	GM-CSF	Granulocyte-macrophage colony-stimulating factor
38	PDGF-AA	Platelet-Derived Growth Factor-AA
39	PDGFAB-BB	Platelet-Derived Growth Factor-BB
40	VEGF	Vascular endothelial growth factor

### Statistical analysis

Data are expressed as mean ± standard deviation (SD). Statistical analysis was performed using Student t test and the Mann-Whitney rank sum test using SigmaStat version 3.5. Scatter plots were performed using Graphpad Prism version 6.00 (GraphPad Software, San Diego California USA). Spearman’s Rho calculator (http://www.socscistatistics.com/tests/spearman/Default2.aspx) was used to check correlation between cytokines levels and visual acuity. For all statistical analyses, a *P* value < 0.05 was considered statistically significant unless mentioned otherwise.

### Heat map cluster analysis

Heatmap were generated using the gplots package in R version 2.15.2. based on differentially expressed cytokines identified through t-test. Where sample values were undetectable below the threshold, the lowest detectable level was assigned; and where concentrations were greater than the range available for analysis, they were assigned the upper limit detection value.

### Principal component analysis

PCoA clustering was performed to observe possible different cytokine profiles between control and endophthalmitis cases of vitreous samples. Cytokine values were log-transformed and subjected to Wilcoxon signed rank test to identify the cytokines, which were differentially expressed in control, culture positive and culture negative samples (Benjamini Hochberg (BH) corrected P < 0.05). PCoA plots were generated (using R v3.2.5, ade4 package) based on differentially expressed cytokines identified through Wilcoxon test (BH corrected P < 0.05), using the Euclidean method. A K-medoids clustering (k = 3) was performed and the samples adhering to three identified clusters were indicated on the PCoA plot.

### Ethical statement

This study adhered to the ARVO statement on human subjects and was approved by the Institutional Review Board of the LV Prasad Eye Institute, Hyderabad, India, and all of the procedures were performed according to the principles of the Declaration of Helsinki.

## Results

The study included 46 patients clinically diagnosed as infectious endophthalmitis and 20 controls who underwent vitrectomy during the same time period. The average age of the patients was 39.30 ± 20.56 years and these comprised of 18-post operative, 25 post traumatic and 3 endogenous endophthalmitis cases. The control group included 20 vitreous samples collected from patients who underwent vitrectomy, for diabetic retinopathy or retinal detachment.

### Microbiology

Of the 46 vitreous samples from patients with infectious endophthalmitis, 25 (54.3%) were culture positive for bacteria (22) or fungi (3) by routine microbiological work-up. The details of these are given in Table 2.

**Table 2:** Microbiological and Demographic details of the patients with presumed infectious endophthalmitis included in the study group.

	Culture positive (25)	Culture negative (21)
**Demographic characteristics**		
Age in years (mean;range)	19.86; 5–75	21.86; 5–82
Sex (male:female)	18:7	11:14
**Diagnosis**		
Traumatic	16	9
Post-operative	8	10
Endogenous	1	2
**Initial Visual acuity**		
Eviseration/Phthisis	0	0
< (20/200)	24	18
> (20/20)—(20/200) <	1	3
= (20/20)	0	0
**Microbiology**		
Bacteria	*Streptococcus pneumoniae* (4)	
	*Enterococcus casseliflavus* (1)	
	*Staphylococcus hominis* (1)	
	*Corynebacterium pseudodiphtheriticum* (2)	
	*Staphylococcus aureus* (2)	
	*Staphylococcus epidermidis* (5)	
	*Achrobacter xylosoxidans* (1)	
	*Enterococcus faecium* (1)	
	*Paenibacillus alvei* (1)	
	*Streptococcus mitis* (1)	
	*Sphingomonas paucimobilis* (1)	
	*Enterobacter cloacae* (1)	
	*Mycobacterium abscessus (1)*	
Fungi	*Cladosporium sp*. (1)	
	*Fusarium solani* (1)	
	*Candida albicans* (1)	

### Association of inflammatory cytokines and chemokines with endophthalmitis

**Fig 1:**
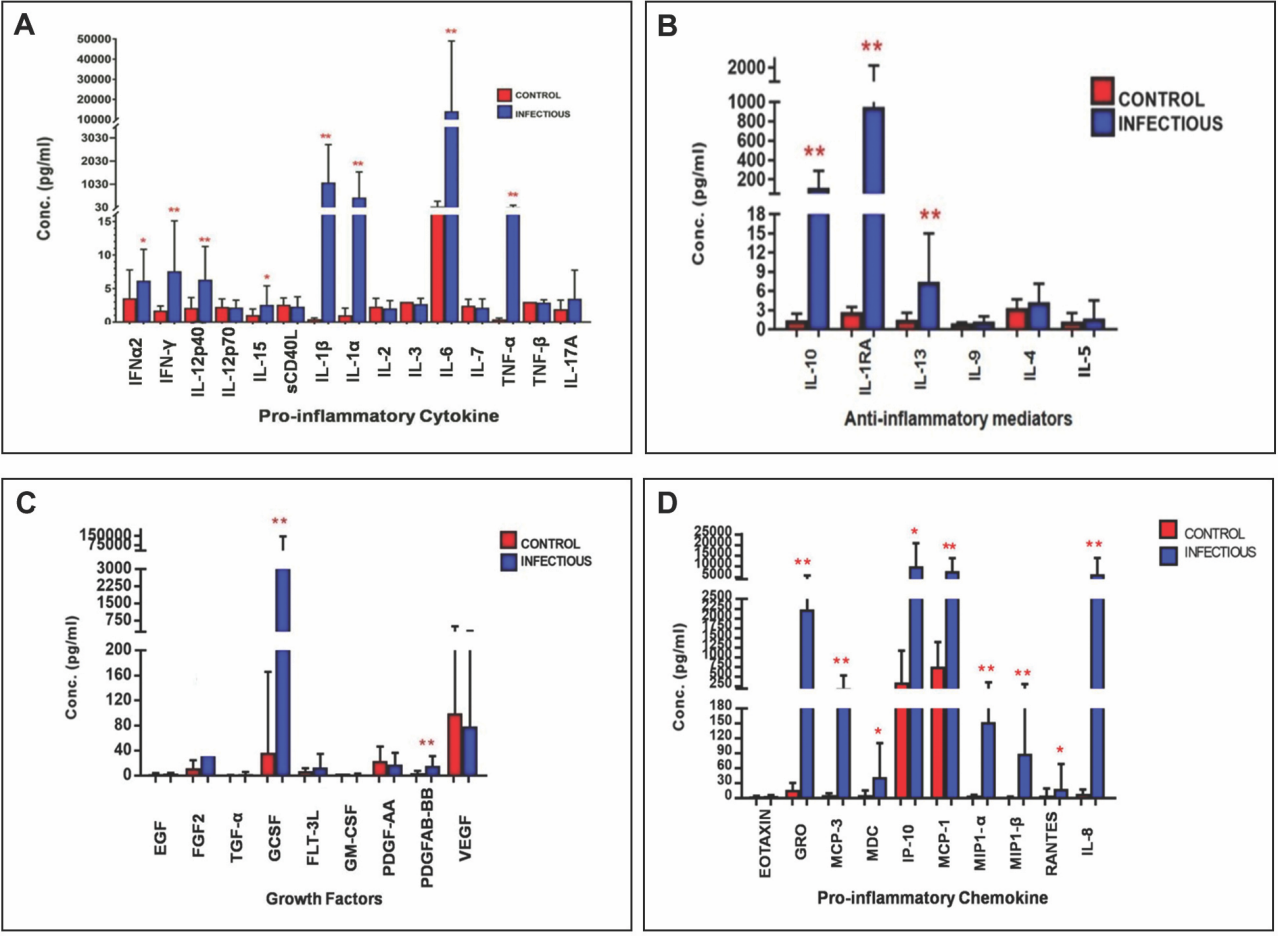
Immune mediator expression in vitreous samples from patients with culture positive infectious endophthalmitis (n = 25) and controls (n = 20). IFNα2—Interferon alpha-2, IFN-γ- Interferon gamma, IL-12p40- interleukin-12 subunit p40, IL-12p70- interleukin-12 subunit p70, IL-15- Interleukin-15, sCD40L- Soluble CD40-ligand, IL-17A- Interleukin-17A, IL-1α - Interleukin 1 alpha, IL-1β - Interleukin 1 beta, IL-2- Interleukin-2,IL-3- Interleukin-3, IL-6- Interleukin-6, IL-7- Interleukin-7, TNFα-Tumor necrosis factor alpha, TNF-β-Tumor necrosis factor beta, Eotaxin- Eotaxin, GRO- Growth-regulated Oncogene, MCP-1- Monocyte chemoattractant protein-1, MCP3- Monocyte chemotactic protein-3, RANTES- Regulated on Activation, Normal T Cell Expressed and Secreted, IL-8- Interleukin-8, IL-10- Interleukin-10, IL-13- Interleukin-13, IL-1RA- Interleukin-1 receptor antagonist, IL-9- Interleukin-9, IL-4- Interleukin-4, IL-5- Interleukin-5, EGF- Epidermal growth factor, FGF2- Fibroblast Growth Factor 2, TGF-α- Transforming growth factor alpha, G-CSF- Granulocyte colony stimulating factor, FLT3L- FMS-like tyrosine kinase 3 ligand, GM-CSF- Granulocyte-macrophage colony-stimulating factor, PDGF-AA- Platelet-Derived Growth Factor-AA, PDGFAB-BB- Platelet-Derived Growth Factor-BB, VEGF- Vascular endothelial growth factor, MDC- Macrophage-derived chemokine, IP-10- Interferon gamma-induced protein 10, MIP-1β- Macrophage Inflammatory Proteins 1 beta, MIP-1α- Macrophage Inflammatory Proteins 1 alpha. Data are represented as mean±SD *p<0.05.

We first wanted to test whether the vitreous of eyes with proven endophthalmitis (culture positive) differs in the levels of chemokines and cytokines with that of control eyes. In each sample, 40 immune mediators were analyzed: 15 pro-inflammatory cytokines (IFN α2, IFNγ, IL12-P40, IL12-P70, IL15, SCD40L, IL-17a, IL-1α, IL-1β, IL-2, IL-3, IL-6, IL-7, TNF-α, TNF- β) and 10 pro-inflammatory chemokines (Eotaxin, GRO, MCP-1, MCP-3, MDC, IP-10, MIP-1β, MIP-1α, Rantes, IL8), 6 anti-inflammatory mediators(IL-10, IL-13, IL-1Ra, IL-9, IL-4, IL-5) and 9 Growth factors(EGF, FGF-2, TGFα, GCSF, FLT3L, GMCSF, PDGF-AA, PDGFAB-BB,VEGF). Among all the 40 immune mediators, 16 mediators [IFN γ (p = 0.000), IL12-P40 (p = 0.001), IL-1α (p = 0.000), IL-1β (p = 0.000), IL-6 (p = 0.000), TNFα (p = 0.000), GRO (p = p = 0.000), MCP-3 (p = 0.000), IL-8 (p = 0.000), MCP-1 (p = 0.000), MIP-1α (p = 0.000), MIP-1β (p = p = 0.000), IL-10 (p = 0.000), IL-13 (p = 0.001), IL-1RA (p = 0.000)], IP-10 (p = 0.008)] were significantly elevated when compared to all controls as shown in (Fig 1A–1D). There was additionally elevated expression of 4 Growth factors including FGF2 (p = 0.000), TGFα (p = 0.000), GCSF (p = 0.000), PDGFAB.BB (p = 0.000), in patients with endophthalmitis when compared to all controls as shown in (Fig 1A–1D). Eight of them (s-CD-40L (p = 0.077), IL12p70 (p = 0.769), IL-2 (p = 0.288), IL-3 (p = 0.119), IL-7(p = 0.203), TNFβ (p = 0.395), PDGF-AA (p = 0.073),VEGF (p = 0.209)) showed statistically not significant downregulation from the control patients.

### Classification analysis

**Fig 2:**
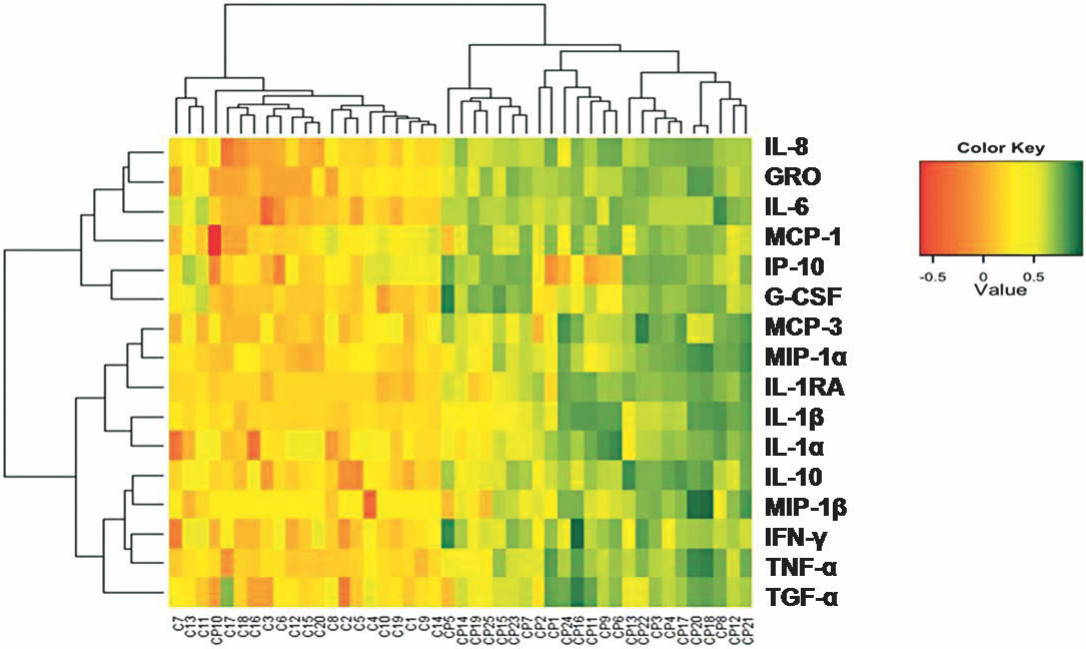
Heat map showing immune mediators concentrations in vitreous of patients with culture positive endophthalmitis (CP), and controls (C), indicated at the *bottom* of the heat map. Color codes in each panel refer to *red* for low expression and *green* for the highest expression levels.

We observed a wide variation in concentrations within the patients with endophthalmitis sample group for multiple cytokines, including MCP-3, GRO, IL-6, IL-8, IP-10, MCP-1α, IL-1α and IL-1ß. Consequently, it was difficult to differentiate patients based on the expression of any one cytokine. To determine whether patients could be better distinguished by global patterns of cytokine expression, heat map analysis was performed for the 16 significant immune mediators to separate the endophthalmitis from the control group and this was confirmed by a decision tree analysis. Presentation of the concentrations of the 16 significant immune mediators in the form of a color-coded heat map which was generated using unsupervised hierarchical clustering gave a good overview of the profile differences between the two groups as shown in Fig 2. The accompanying dendrogram can be divided into two principle clusters that largely segregate into non-infectious controls and patients with endophthalmitis.

### Biomarker Identification of inflammatory mediators in Vitreous of patients with culture negative endophthalmitis

**Fig 3:**
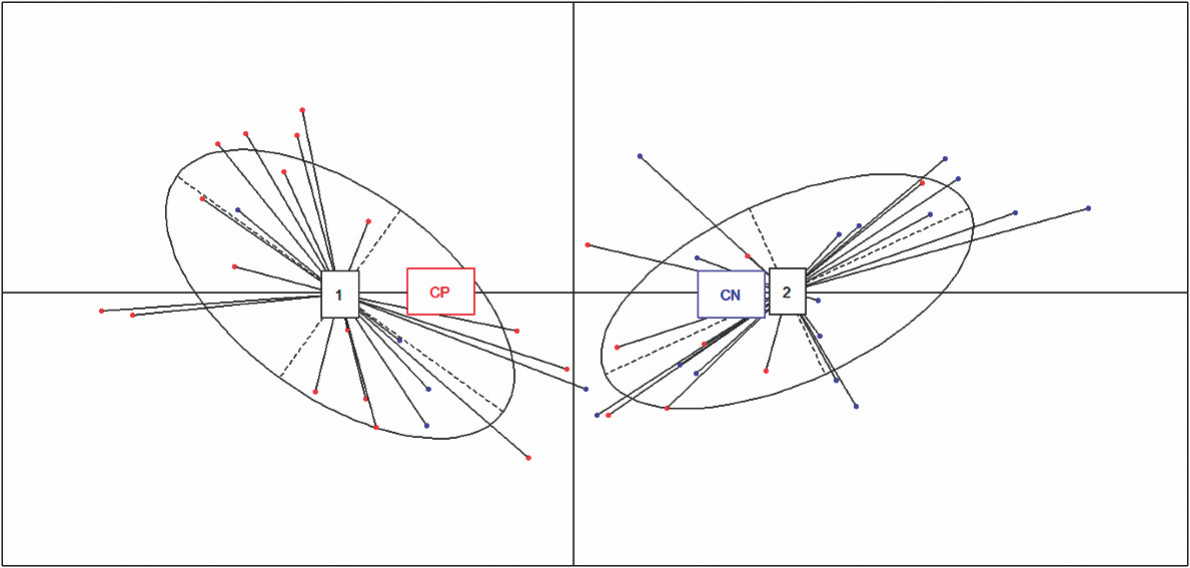
PCoA plot of all culture positive (CP) and culture negative(CN) vitreous samples showing two components using the 16 differentially expressed biomarkers. The analysis depicts that cytokines from the CP and CN groups overlapped, and could not be distinguished.

To assess which parameters may be applicable as biomarkers for the diagnosis of presumed infectious endophthalmitis, we checked the expression level of 16 significant immune mediators in patients with culture negative endophthalmitis by PCoA plot (Fig 3). A comparison of these cytokines in the vitreous between patients with culture positive (CP) endophthalmitis (n = 25) and culture negative endophthalmitis (CN) (n = 21) showed mixed clusters which indicates that CP and CN samples have similar cytokine and chemokine profiles that are not distinctly separable from each other. All 16 cytokines displayed comparable levels between 2 groups.

**Fig 4:**
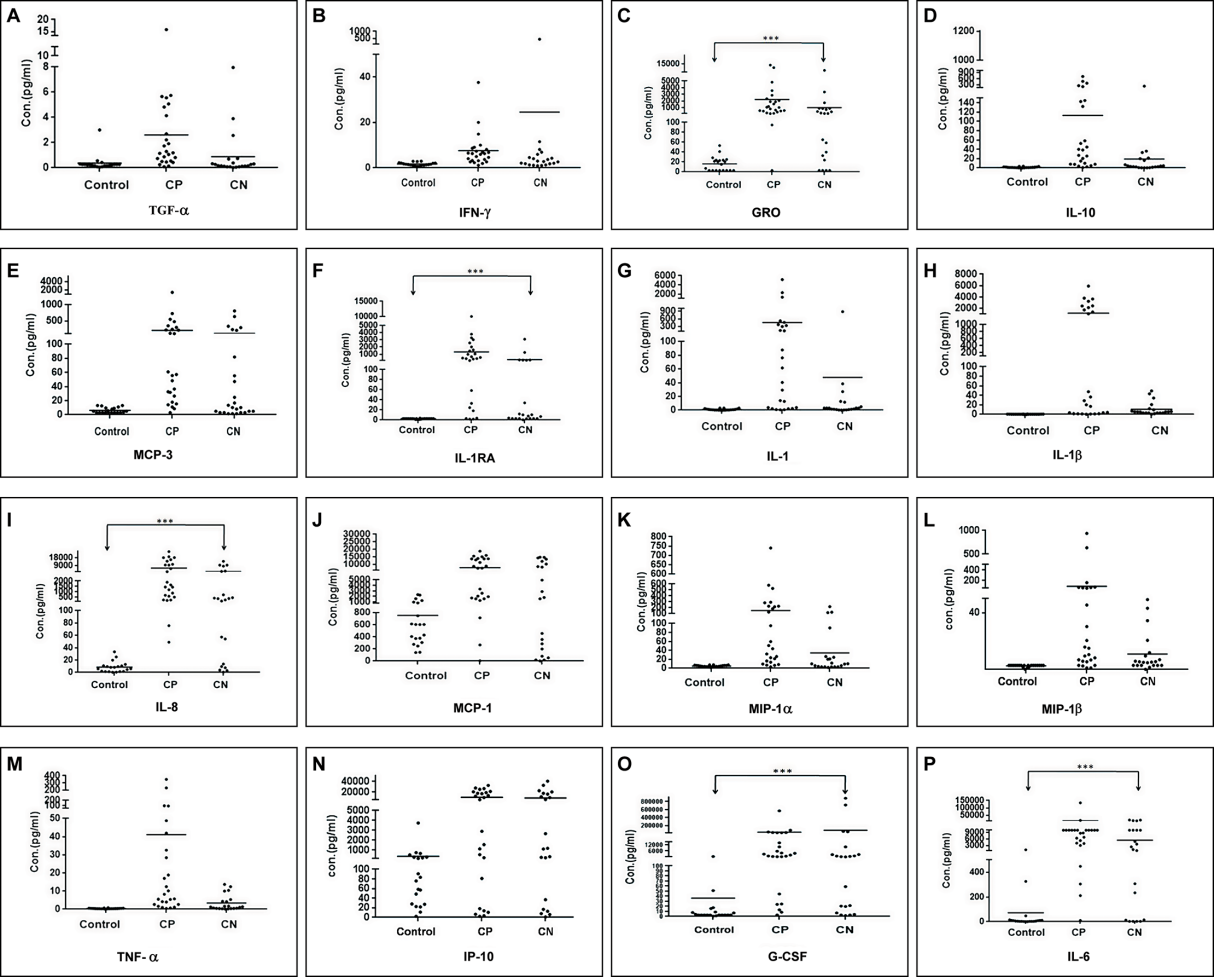
Scatter plot graph of the concentrations (pg ml−1) of the 16 significant immune mediators in all patient samples collected in the study including controls, CP (culture positive endophthalmitis) and CN (culture negative endophthalmitis). (a) TGF-α (b) IFN-γ; (c) GRO; (d) IL-10; (e) MCP3 (f) IL-1RA (g) IL-1α (h) IL-1β (i) IL-8(j) MCP-1 (k) MIP-1α (l) MIP-1β (m) TNFα (n) IP-10 (o) G-CSF (p) IL-6. Data are represented as mean±SD ***p<0.001.

In a next step, to assess which parameters could be applicable as biomarkers for the diagnosis of endophthalmitis scatter plots were generated for each of these 16 immune mediators as shown in Fig 4, displaying the distribution of values in all groups. Comparing the cytokines and chemokines contributing to the separation between the culture negative (CN) endophthalmitis group and the control group, five mediators were identified namely, GRO, IL-6, IL-8, G-CSF and IL-1 RA alpha which exhibited significant differences among the concentrations with minimal overlap between groups. These differences in expression might denote the potential targeted biomarkers for differentiating infectious endophthalmitis from non-infectious conditions.

### Cluster analysis identifies a distinct group of inflammatory cytokines and chemokines that could be independent predictors of disease

**Fig 5:**
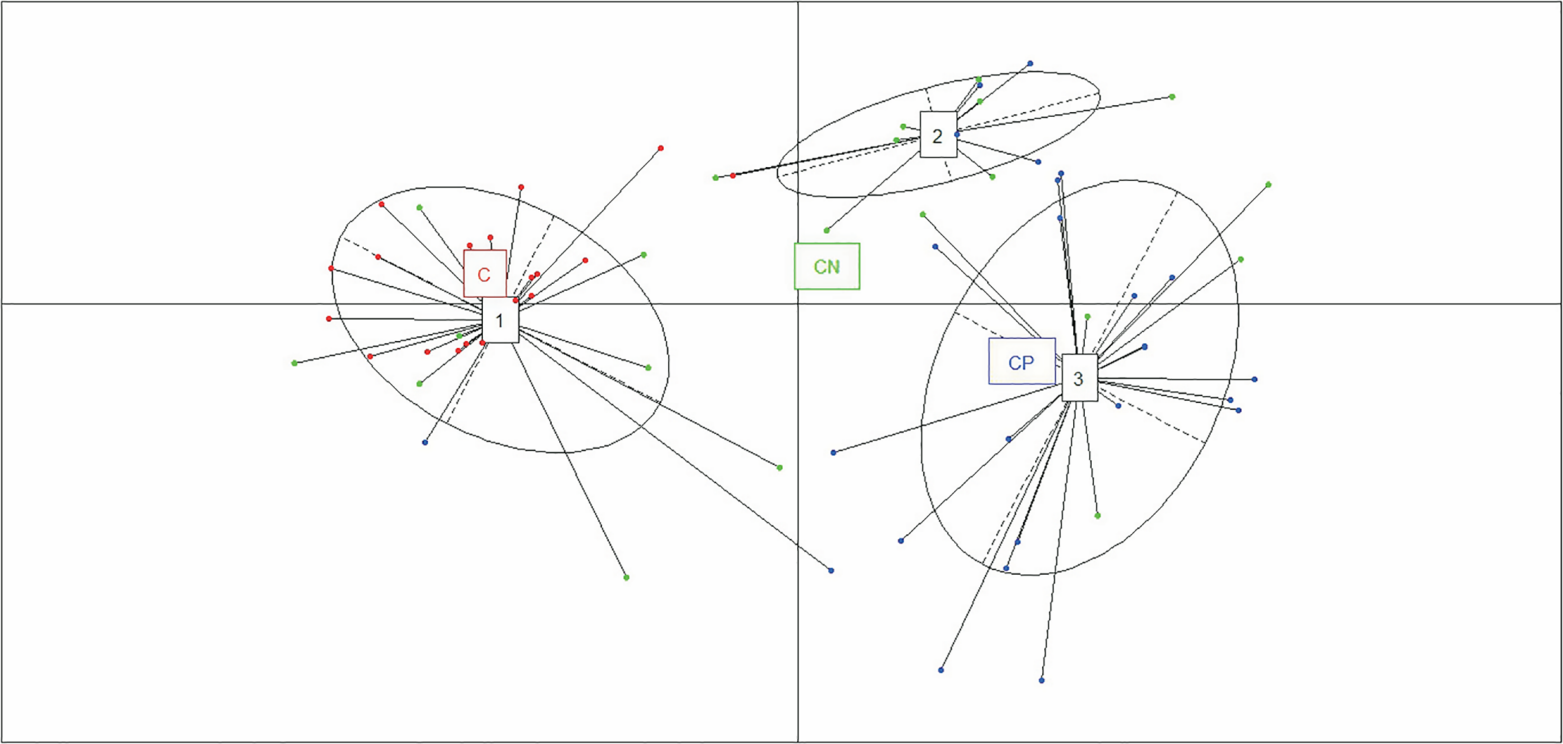
PCoA plot of all culture positive (CP) and culture negative(CN) endophthalmitis samples along with controls showing two primary components using the 5 differentially expressed biomarkers. The analysis depicts that cytokines form three clusters that could be easily distinguished with minimal overlap.

Using our five-biomarker signature, we aimed to distinguish patients in the three clinical groups (CP vs. CN vs. uninfected controls). To confirm this, we applied cluster analysis by PCoA plot to understand the pattern of variation among the groups based on the expression profiles of the five mediators identified. Application of this method to our data showed that the samples could be divided into three principal groups: one consisting of controls (Fig 5, cluster 1) and two independent clusters of patients with endophthalmitis (Fig 5, clusters 2 and 3). While the CP samples primarily clustered together in Cluster 3, the CN samples though distinguishable, clustered with Cluster 1 and Cluster 3 along with C and CP group respectively. This indicates that some CN samples have same cytokine profiles of Control groups, while other share similarity with CP groups. Interestingly, four CP samples (16%) clustered with CN group in cluster 2 while two CP samples (8%) clustered with Control group. The two CP samples which clustered with control group grew fungal organisms in microbiology culture. Thus Principal component analysis data showed that infectious endophthalmitis samples appear to contain different cytokines when compared to the non-infectious conditions, as these segregated from the controls on the first principal component (Fig 5). Cluster analysis further showed that the three clusters segregate primarily by the expression of a subset of cytokines, ranging from low, intermediate, and high levels in clusters 1(C), 2(CN), and 3(CP), respectively. The smaller spread of the C cluster indicates that there was lesser variation in the expression levels of selected five immune mediators in the control samples. Comparatively, the samples in the CP cluster showed higher variation among the samples resulting in a relatively bigger spread of the CP cluster. This observation reiterates the unique cytokine profile of Control samples compared to infectious endophthalmitis samples and these five mediators could thus be used as independent predictors of disease.

### Correlation of cytokines with visual acuity

Visual acuity was analyzed with LogMAR values and correlated to the 16 significant vitreal cytokine values by pairwise Spearman’s rank coefficients (data in S1 Table). Spearman’s rank coefficients analyses showed a significant correlation between the initial visual acuity and intraocular levels of TGF-α, IL-1β and IL-8. There was no significant correlation between any of the cytokines and final visual acuity and also with the severity and visual outcome (data in S1 Table).

## Discussion

The clinical presentation of endophthalmitis can vary widely, and despite early therapeutic and/or surgical intervention, can lead to complete vision loss or potentially loss of the eye itself. The ability of an organism to cause endophthalmitis is not only related to the virulence of the organism but also to the load of the invading organims and the production of toxins that stimulate the inflammatory cascade. Cytokines are produced by the immune system in response to invading pathogens [8]. A network of cytokine signals is essential in modulation of the inflammatory response, clearance of pathogens, and subsequent repair of infected tissues. Foxman and colleagues [10] have demonstrated the involvement of many cytokines and chemokines in intraocular inflammation in uveitis and suggested that these mediators could be an attractive target for immune therapy. More recently, immunomodulatory agents targeting TNF-α have been approved for the treatment of Crohn's disease, rheumatoid arthritis and juvenile chronic arthritis [11]. Many others are being developed in treatment of other chronic inflammatory diseases. Several reports have demonstrated elevated intraocular cytokine levels in various human ocular inflammatory conditions including endophthalmitis [4,5,7–8,10], however culture negative endophthalmitis as a clinical entity was not considered in these studies. Cytokine profiling of patients with endophthalmitis may represent a valuable tool for delineating the infectious endophthalmitis from sterile endophthalmitis, thus allowing the initiation of appropriate treatment, especially in culture negative cases. In the present study, a multiplex analysis of vitreal cytokines in patients with CP and CN endophthalmitis was able to identify cytokine profiles associated with the disease, as well as visual function. While the expression of cytokines in patients with endophthalmitis in our study were mostly consistent with the previous reports [5,8,12], Sauer et al [5]. also reported an increase in VEGF levels which was not seen on our study. Also we did not observe an increase in PDGF-BB, RANTES and IL17 as reported by Escarião et al [12]. While our control samples include DR and RD subjects and it is possible that the overall cytokines/chemokines in controls was lower than DR conditions alone. However, our focus was infectious versus non-infectious inflammatory conditions, therefore, we went ahead with comparison with a combined data of the two control groups. In this study, we observed a consistent significant difference in the expression of 16 cytokines and chemokines among the patients with endophthalmitis compared to the non-infectious controls.

Obviously, in endophthalmitis an immune response is mounted which generates cell activation and cytokine secretion in order to suppress the infectious process. In our study, this increase was particulary noticeable for proinflammatory cytokines such as IFN-γ, IL-1α, IL-1β, IL-6, TNFα and chemokines such as MCP3, IP-10, MCP-1, MIP-1α, MIP-1β, IL-8, GRO. Additionally, anti-inflammatory cytokines such as IL-10 and IL-1RA were also significantly upregulated in infectious endophthalmitis. These cytokines are mainly involved in all phases of the immune response, including: recognition (mainly IL-1), recruitment of leukocytes (IL-8), pathogen removal by the activation of macrophages (IFN-γ and chemokines) and lymphocytes (IL-2 and IL-6) and tissue repair (growth factors). Yu. et al [13]. reported that IL-6 is an important pro-inflammatory cytokine, elevated in the sera of patients with inflammatory diseases; therefore, although our samples were from a different source (vitreous), our results appear to be consistent with their findings.

In this study, we additionally tried to evaluate if there was any specific cytokine network related to the type of identified pathogens and we observed a significant upregulation in 15/16 cytokines (IFN-γ, GRO, IL-10, MCP3, IL-1RA, IL-1α, IL-1β, IL-8, MCP-1, MIP-1α, MIP-1β, TNFα, IP-10, G-CSF, IL-6 in cases of gram positive infections (data in S2 Table). We could not determine the cytokines involved in gram negative infections, probably because we had only three cases of these infections and the numbers were not sufficient for a correlation analysis. Moreover, the ability of bacteria to cause endophthalmitis may also be related to the intraocular bacterial load and production of toxins. Unfortunately, it is not possible to measure bacterial load in a classical clinical follow-up. Interestingly, in the three cases of fungal endophthalmitis included in our study, we observed a significant upregulation of IL-1β and TNFα and TGF-α, of which TGF-α, is the only cytokine to be downregulated in bacterial endophthalmitis (data in S2 Table). In case of fungal endophthalmitis the cytokine profile correlated with the clinical observation of reduced inflammation compared to bacterial endophthalmitis. The concentration of the cytokines in the vitreous from patients with fungal endophthalmitis was closer to the non-infectious controls (Fig 2, #CP10, #CP14 and #CP19).

Culture-negative endophthalmitis (CN) is a challenging clinical entity, both diagnostically and therapeutically [1,3]. Significantly increased expression of IL-1RA, IL-6, IL-8, GRO, G-CSF was observed in the vitreous from patients with culture negative but presumed infectious endophthalmitis compared to the non-infectious controls and a cluster analysis of these cytokines distinguished the patients with endophthalmitis from the control group, thus providing us a five-biomarker signature. Though we might use this biomarker signature for differentiating culture negative endophthalmitis from sterile endophthalmitis, we were unable to identify a biomarker signature with sufficient discriminatory power for the type of microorganism that might be involved. A major limitation in the study is that we were unable to validate these biomarker with a single—target ELISAs, for lack of sample and our future studies would provide the clinical utility of these biomarkers. In our study, we also tried to link visual acuity (at initial presentation and at final follow-up) and cytokines levels and we observed a significant correlation between the visual acuity at admission and the intraocular levels of cytokines TGF-α, IL-1β and IL-8. It may be too early to predict which of these new biomarkers will be useful clinically. There is little information in the literature regarding the time course of these changes, their ability to predict visual outcome, and how effectively they may be utilized in support of novel therapies targeting these co-stimulatory molecules. A well-designed animal study with time dependent analysis may provide the answer. In near future, larger scale studies might be required to confirm present results because the number of samples was small in the present study. To conclude, our study provides a new means for improving the diagnostic yield of endophthalmitis and with the identification of specific targets such as inflammatory cytokines and growth factors, new therapeutic approaches may be of interest in the future.

## Supporting information

S1 Table Spearman’s rank coefficient analyses of the association between the 16 significant intraocular cytokines levels and visual acuity at admission and at final follow-up. (DOCX)

S2 Table Mann-Whitney correlation of cytokines and mean cytokine expression of proven culture positive endophthalmitis. (DOCX)
