# Supplementary material for: Elevated cytokine levels in vitreous as biomarkers of disease severity in infectious endophthalmitis
Source: PLoS One. 2018 Oct 8;13(10):e0205292. doi: 10.1371/journal.pone.0205292 (PMC6175518; doi:10.1371/journal.pone.0205292)
Supplement: S1 Table — (DOCX) [file pone.0205292.s001.docx]

S1_TABLE : Spearman’s rank coefficient analyses of the association between the 16 significant intraocular cytokines levels and visual acuity at admission and at final follow-up.

| **S. No.** | **Cytokines** | **Initial Visual Acuity** | |  | **Final Visual Acuity** | |
| --- | --- | --- | --- | --- | --- | --- |
|  |  | **p-value** | **r-value** |  | **p-value** | **r-value** |
| 1 | **TGF-α** | **P is 0.03966** | **R is 0.30446** |  | P is 0.14808 | R is 0.21667 |
| 2 | IFN-γ | P is 0.38605 | R is 0.13085 |  | P is 0.86415 | R is -0.02594 |
| 3 | GRO | P is 0.27766 | R is 0.16348 |  | P is 0.85184 | R is -0.02831 |
| 4 | IL-10 | P is 0.38177 | R is 0.13202 |  | P is 0.76855 | R is -0.04459 |
| 5 | MCP3 | P is 0.36652 | R is 0.13627 |  | P is 0.94834 | R is 0.00982 |
| 6 | IL-1RA | P is 0.10822 | R is -0.23996 |  | P is 0.10615 | R is -0.24133 |
| 7 | IL-1α | P is 0.1649 | R is 0.20824 |  | P is 0.78314 | R is 0.04171 |
| 8 | **IL-1β** | **P is 0.04534** | **R is 0.29659** |  | P is 0.28298 | R is 0.1617 |
| 9 | **IL-8** | **P is 0.03197** | **R is 0.31675** |  | P is 0.63922 | R is 0.07099 |
| 10 | MCP-1 | P is 0.94068 | R is -0.01128 |  | P is 0.4019 | R is -0.12658 |
| 11 | MIP-1α | P is 0.96394 | R is 0.00686 |  | P is 0.52316 | R is -0.09658 |
| 12 | MIP-1β | P is 0.41918 | R is 0.12202 |  | P is 0.81788 | R is -0.0349 |
| 13 | TNFα | P is 0.53502 | R is 0.09385 |  | P is 0.94919 | R is -0.00966 |
| 14 | IP-10 | P is 0.07253 | R is -0.26728 |  | P is 0.05617 | R is -0.28357 |
| 15 | G-CSF | P is 0.79862 | R is -0.03867 |  | P is 0.1429 | R is -0.21941 |
| 16 | IL-6 | P is 0.37238 | R is 0.13463 |  | P is 0.29692 | R is -0.15716 |

Statistical significant values (p < 0.05) appear in bold.
