# Supplementary material for: Elevated cytokine levels in vitreous as biomarkers of disease severity in infectious endophthalmitis
Source: PLoS One. 2018 Oct 8;13(10):e0205292. doi: 10.1371/journal.pone.0205292 (PMC6175518; doi:10.1371/journal.pone.0205292)
Supplement: S2 Table — (DOCX) [file pone.0205292.s002.docx]

S2_Table: Mann-Whitney correlation of cytokines and mean cytokine expression of proven culture positive endophthalmitis

|  | **Gram Positive** |  | **Gram Negative** |  | **Fungus** |
| --- | --- | --- | --- | --- | --- |
|  | **P-value** |  | **P-value** |  | **P-value** |
| **TGF-α** | P = 0.839 |  | P = 0.824 |  | **P = 0.001** |
| **IFN-γ** | **P = <0.001** |  | P = 0.100 |  | P = 0.679 |
| **GRO** | **P = <0.001** |  | P = 0.100 |  | P = 0.100 |
| **IL-10** | **P = <0.001** |  | P = 0.002 |  | P = 0.563 |
| **MCP3** | **P = <0.001** |  | P = 0.100 |  | P = 0.100 |
| **IL-1RA** | **P = <0.001** |  | P = 0.100 |  | P = 0.700 |
| **IL-1α** | **P = <0.001** |  | P = 0.100 |  | P = 0.187 |
| **IL-1β** | **P = <0.001** |  | P = 0.400 |  | **P = 0.001** |
| **IL-8** | **P = <0.001** |  | P = 0.100 |  | P = 0.100 |
| **MCP-1** | **P = <0.001** |  | P = 0.100 |  | P = 0.100 |
| **MIP-1α** | **P = <0.001** |  | P = 0.100 |  | P = 0.100 |
| **MIP-1β** | **P = <0.001** |  | P = 0.220 |  | P = 0.338 |
| **TNFα** | **P = <0.001** |  | P = 0.191 |  | **P = 0.001** |
| **IP-10** | **P = <0.001** |  | P = 0.100 |  | P = 0.700 |
| **G-CSF** | **P = <0.001** |  | P = 0.100 |  | P = 0.100 |
| **IL-6** | **P = <0.001** |  | P = 0.100 |  | P = 0.100 |

Statistical significant values (p < 0.001) appear in bold.
